# Incidence, Mortality, and Federal Research Funding by Cancer Type in the US

**DOI:** 10.1001/jamanetworkopen.2026.7837

**Published:** 2026-04-20

**Authors:** Chirayu Mohindroo, Anish Thomas

**Affiliations:** 1 National Cancer Institute, National Institutes of Health, Bethesda, Maryland

## Abstract

This cross-sectional study examines the association between disease burden and US federal funding of cancer research in fiscal year 2025.

## Introduction

Both cancer outcomes and the distribution of federal research funding across cancer types continue to evolve. Federal funding reflects a combination of peer-reviewed scientific opportunity, historical investment, and congressional appropriations. Assessing how current funding aligns with contemporary measures of disease burden may help identify gaps. Incidence alone provides an incomplete measure of public health impact because it does not capture disease lethality. Mortality, survival, and mortality-to-incidence ratios (MIRs) offer complementary perspectives and more accurately reflect clinical urgency and unmet need. We examined the current US federal cancer research funding landscape in relation to disease burden across major cancer types. We hypothesized that the association between federal funding and disease burden would vary depending on outcome-based measures.

## Methods

In this cross-sectional study, we analyzed national cancer registry data on incidence and 5-year survival together with US National Institutes of Health (NIH) funding across selected major cancer types.^1,2^ Incidence and survival were obtained from the Surveillance, Epidemiology, and End Results Program (21 registries, 2015-2021) and the North American Association of Central Cancer Registries’ Cancer in North America Explorer (2022). Fiscal year 2025 NIH funding data were extracted from public NIH portfolio reports. Mortality was approximated by multiplying annual incident cases by the proportion not surviving to 5 years. Funding per incident case, per estimated death, and mortality-to-incidence ratios (MIRs) were calculated. RStudio Desktop version 2025.09.1 was used for this analysis (Posit team, 2025).

This study followed the STROBE reporting guideline. This study used publicly available, deidentified, aggregate data and therefore did not constitute human participants research under the Common Rule (45 CFR 46.102); institutional review board approval and informed consent were not required.

## Results

The analysis included 9 major cancer types in the US with substantial variation in incidence, survival, estimated mortality, and NIH research funding (Table, Figure). Lung cancers (small cell [SCLC] and non–small cell [NSCLC]) caused 151 401 deaths. Pancreatic cancer caused 49 211 deaths; breast cancer, 22 606; and prostate cancer, 5219 deaths. When evaluated using MIR, these patterns were more pronounced: MIRs exceeded 0.85 for SCLC and pancreatic cancer, indicating that most diagnoses result in death, and were below 0.10 for breast and prostate cancers. National Cancer Institute research funding totaled $1.58 billion for breast cancer, $62 million for SCLC, $227 million for NSCLC, and $440 million for pancreatic cancer. On a per estimated death basis, funding was $69 800 for breast cancer and $126 992 for prostate cancer, vs $2818 for SCLC and $8945 for pancreatic cancer.^3^

**Table.** Incidence, Survival, Mortality, and NIH Research Funding Across Selected Cancer Types in the US

Cancer types	5-y survival, %^a^	No. of cases^b^	Age-standardized incidence rates (SE)^b^	Funding for all active NIH projects, $^c^	Estimated deaths	Funding per death, $	Funding per incidence, $	Mortality-to-incidence ratio
Small cell lung cancer	9.1 (8.8-9.5)	24 466	5.6 (0.04)	62 677 595	22 240	2818	2562	0.909
Non–small cell lung cancer	30.8 (30.6-31.0)	186 649	43.5 (0.10)	226 588 717	129 161	1754	1214	0.692
Colorectal cancer	65.4 (65.1-65.6)	143 282	36.6 (0.10)	494 722 934	49 576	9979	3453	0.346
Pancreatic cancer	13.3 (13.0-13.5)	56 760	13.6 (0.06)	440 205 032	49 211	8945	7756	0.867
Breast cancer (female)^d^	91.7 (91.5-91.8)	272 361	133.3 (0.27)	1 577 894 193	22 606	69 800	5793	0.083
Prostate cancer^d^	97.9 (97.8-98.1)	248 541	119.4 (0.24)	662 815 455	5219	126 992	2667	0.021
Ovarian cancer^d^	51.6 (51.1-52.2)	20 046	9.9 (0.07)	419 863 272	9702	43 275	20 945	0.484
Liver cancer	22.0 (21.6-22.3)	35 661	8.3 (0.04)	290 580 634	27 816	10 447	8148	0.780
Stomach cancer	37.9 (37.4-38.4)	27 346	6.8 (0.04)	104 861 823	16 982	6175	3835	0.621

Abbreviation: NIH, National Institutes of Health.

^a^ Based on the Surveillance, Epidemiology, and End Results Program 21 registries, 2015-2021, all ages and overall.

^b^ Based on the North American Association of Central Cancer Registries database, 2022, all ages. All states (excluding Indiana) and Washington, DC, are included.

^c^ Funding for all active NIH projects for fiscal year 2025 as of August 1, 2025.

^d^ Incidence rates for sex-specific cancers were calculated only among the corresponding sex.

**Figure.**
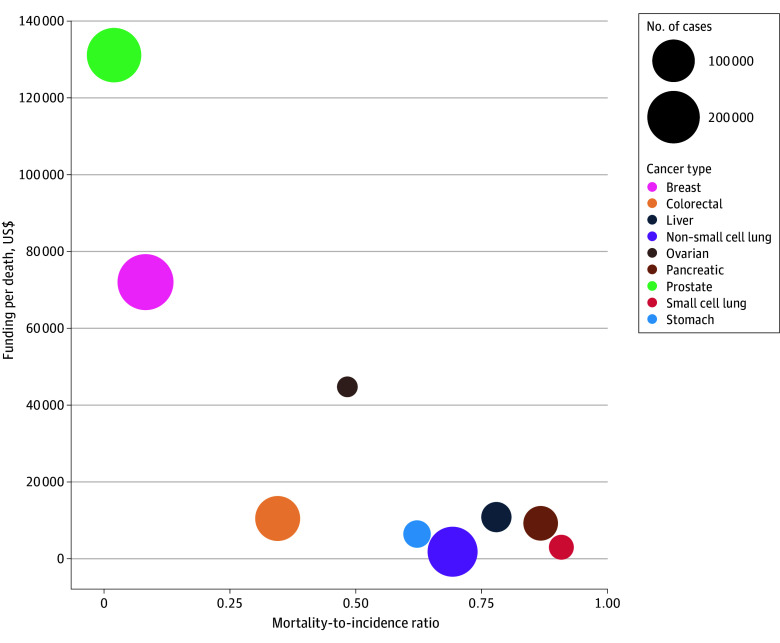
Association Between Estimated Annual Cancer-Related Deaths and National Institutes of Health (NIH) Research Funding Across Selected Cancer Types in the US Scatter plot illustrating the association between estimated annual deaths, derived from cancer incidence data from the Cancer in North America Explorer of the North American Association of Central Cancer Registries (2022) and 5-year survival estimates from the Surveillance, Epidemiology, and End Results Program 21 registries (2015-2021), and total annual NIH research funding for active projects in fiscal year 2025. The x-axis reflects the mortality-to-incidence ratio, and the y-axis shows funding per estimated death. Bubble size represents cancer incidence.

## Discussion

These findings indicate that cancers with the highest lethality receive disproportionately lower levels of federal research support. Prioritizing these cancers could help direct limited resources toward diseases with the greatest potential to reduce suffering, particularly as outcomes continue to improve for less lethal malignant neoplasms. Beyond mortality, prioritization should also account for the disproportionate impact of certain cancers on underserved populations, quality-of-life burden, and opportunities for prevention. Current funding patterns reflect historical progress in specific cancer types, including prior therapeutic breakthroughs, long-standing research infrastructure, and sustained advocacy-driven investment that emerged following major advances in screening and treatment (eg, breast and prostate cancer).^4^ Cancers with limited advocacy or philanthropic support may depend largely on federal funding, magnifying the effects of funding imbalances, while industry investment, which often tracks incidence rather than lethality, may further reinforce these patterns. Although this misalignment has been recognized for more than a decade,^5^ our findings indicate that funding patterns remain largely unchanged. Limitations include reliance on a single fiscal year of funding data, the use of estimated rather than observed mortality counts, and the lack of granularity regarding funding by research category; nonetheless, these metrics may help identify persistent underinvestment and may help promote data-driven discussions on resource allocation, while recognizing that burden-based measures alone cannot fully capture scientific opportunity, feasibility, or prevention potential. These findings support a composite framework for federal funding that integrates incidence with outcome-based measures such as mortality and survival, alongside consideration of nonfederal research investment from philanthropy and industry, to better align resources with areas of greatest clinical need.

## Notes

Supplement.
Data Sharing Statement
