# Supplementary material for: Incidence, Mortality, and Federal Research Funding by Cancer Type in the US
Source: JAMA Netw Open. 2026 Apr 20;9(4):e267837. doi: 10.1001/jamanetworkopen.2026.7837 (PMC13096976; doi:10.1001/jamanetworkopen.2026.7837)
Supplement: Supplement. — Data Sharing Statement [file jamanetwopen-e267837-s001.pdf]

Data Sharing Statement

Mohindroo. Incidence, Mortality, and Federal Research Funding by Cancer Type in the US. *JAMA Netw Open*. Published April 20, 2026. doi:10.1001/jamanetworkopen.2026.7837

Data
Data available: Yes
Data types: Other (please specify)
Additional Information: All data used in this study are publicly available. Incidence and survival data were obtained from the National Cancer Institute's SEER Program (https://seer.cancer.gov) and the NAACCR Public Use dataset (https://www.naaccr.org). NIH funding data were accessed through the NIH Research Portfolio Online Reporting Tools (RePORTER) database (https://reporter.nih.gov).
How to access data: SEER (Surveillance, Epidemiology, and End Results Program) National Cancer Institute https://seer.cancer.gov NAACCR (North American Association of Central Cancer Registries) Public Use Dataset https://www.naaccr.org NIH Research Portfolio Online Reporting Tools (RePORTER) (Funding for all active NIH projects) https://reporter.nih.gov
When available: With publication

Supporting Documents
Document types: None

Additional Information
Who can access the data: anyone requesting the data
Types of analyses: for any purpose
Mechanisms of data availability: These data can be requested on the above mentioned websites
